# 胸外科术后急性肺栓塞的诊断与治疗——附37例胸外术后急性肺栓塞病例的诊治经验

**DOI:** 10.3779/j.issn.1009-3419.2018.10.07

**Published:** 2018-10-20

**Authors:** 哲 许, 晓溪 范, 顺 许

**Affiliations:** 110001 沈阳，中国医科大学附属第一医院胸外科 Department of Thoracic Surgery, the First Affiliated Hospital of China Medical University, Shenyang 110001, China

**Keywords:** 胸外科手术, 肺栓塞, 肺血栓栓塞症, 静脉血栓栓塞症, Thoracic surgery, Pulmonary embolism, Pulmonary thromboembolism, Venous thromboembolism

## Abstract

**背景与目的:**

肺栓塞（pulmonary embolism, PE）是胸外科术后最严重的并发症之一，因此充分了解胸外科术后急性PE患者所具有的临床特点具有重要意义。本研究通过总结37例胸外科术后急性PE患者的临床特点及诊治体会，从而提高胸外科术后急性PE的预防及诊治水平。

**方法:**

对37例胸外科术后急性PE患者进行回顾性分析，对性别、年龄、体重指数（body mass index, BMI）、诊断及手术术式及术后发生PE时间、临床表现、诊断及治疗过程进行综合分析。

**结果:**

37例患者中男16例（43.2%），女21例（56.8%），平均年龄为（65.64±6.29）岁（53岁-82岁），32例患者年龄超过60岁（86.5%）。BMI范围位于17.1 kg/m^2^-30.8 kg/m^2^之间，中位BMI为26.3 kg/m^2^，27例（73.0%）患者的BMI超过25.0 kg/m^2^。恶性肿瘤34例（91.9%）。中位发病时间为术后第4天，其中发生在术后第3天的患者有11例，所占比例（29.7%）最高。上午9点至晚上9点发生PE的比例可达77.8%。术后D二聚体（D-dimer, D-D）波动在1.0 μg/mL-20.0 μg/mL（FEU）之间，平均值为（7.09±4.45）μg/mL（FEU），其中32例（86.5%）患者的术后D-D高于3.00 μg/mL（FEU）。

**结论:**

充分掌握胸外科术后急性PE患者的临床特点，及早发现诊断并采取多学科治疗能大大提高疾病生存率。

## Abstract (en)

**Background and objective:**

Pulmonary embolism (PE) is one of the most severe complications after thoracic surgeries. Thus it is of great importance to learn the characteristics of acute PE after thoracic surgeries. This study summarized the clinical characteristics and experience on the diagnosis and treatment of 37 patients with postoperative acute pulmonary embolism, in order to improve its prophylaxis and management level.

**Methods:**

We retrospectively reviewed 37 patients with postoperative acute pulmonary embolism following thoracic surgeries. Age, gender, body mass index (BMI), diagnosis, surgical procedure, onset time, clinical presentation, diagnosis and management were comprehensively analyzed.

**Results:**

There were 16 males (43.2%) and 21 females (56.8%). The average age was (65.64±6.29) years (range from 53 years to 82 years) and 32 patients were over 60 years. BMI ranged from 17.1 kg/m^2^ to 30.8 kg/m^2^ with median of 26.3 kg/m^2^. And 27 patients' BMI (73.0%) were over 25.0 kg/m^2^. Thirty-four patients (91.9%) were with malignancies. Median presentation time was the 4th day postoperatively, while 11 patients were presented on the 3^rd^ day postoperatively which accounted for the most. Patients with acute pulmonary embolism accounted for 77.8% from 9 am to 9 pm. D-dimer (D-D) ranged from 1.0 μg/mL-20.0 μg/mL (FEU) with median of (7.09±4.45) μg/mL (FEU) and 32 (86.5%) patients' D-D were over 3.00 μg/mL (FEU).

**Conclusion:**

The survival rate of postoperative acute pulmonary embolism can be increased by fully understanding its clinical characteristics, early diagnosis and multiple disciplinary treatment.

肺栓塞（pulmonary embolism, PE）是以各种栓子阻塞肺动脉系统为发病原因的一组疾病或临床综合征的总称，包括肺血栓栓塞症（pulmonary thromboembolism, PTE）、脂肪栓塞综合征、羊水栓塞、空气栓塞等，其中PTE为PE的最常见类型。作为胸外科术后最严重的并发症之一^[1]^，PE的发生率可以达到5%^[2-4]^，在肺叶切除术后早期死因中仅次于肺炎，排在第二位^[5]^。因此充分了解胸外科术后急性PE患者所具有的临床特点，做到术前及时预判与早期预防具有重要意义。但目前对于胸外科术后PE临床特点及诊治的研究很少，因此，本研究的目的在于对胸外科术后急性PE的病例进行回顾性分析并针对其临床特点进行归纳总结。

## 1 对象与方法

### 1.1 研究对象

2011年-2018年8月期间在我院胸外科住院接受手术的患者11, 722例，其中37例患者于术后出现急性PE，为本研究的入选对象，术后急性PE发病率为0.3%（37/11, 722）。37例患者中29例（78.4%）经积极治疗后病情逐渐好转并最终出院，8例患者虽经积极抢救但病情逐渐恶化并最终死亡，病死率为21.6%（8/37）。诊治成功的29例患者均由随后的CT肺动脉造影（computed tomographic pulmonary angiography, CTPA）检查确诊为肺动脉栓塞，8例死亡病例未进行CTPA检查，为临床诊断，其主要依据患者发病时的临床表现及辅助检查，如发病时患者Wells评分大于6分、D二聚体（D-dimer, D-D）显著增高、以及V1-V4的T波改变和ST段异常的异常心电图表现等。

### 1.2 研究方法和观察指标

通过查阅电子病例分别对这些病例的性别、年龄、体重指数（body mass index, BMI），诊断及手术术式、疾病发生时间、发病前的活动、病情转归、治疗是否应用气管插管及机械辅助通气、是否具有恶性肿瘤病史、D-D、发病时心电图的异常表现进行记录。

### 1.3 统计学方法

使用SPSS 22.0统计分析软件进行数据处理分析。数据的统计描述采用表格、条形图、饼状图及散点图等。

## 2 结果

37例患者中男16例（43.2%），女21例（56.8%），平均年龄为（65.64±6.29）岁（53岁-82岁），超过60岁的患者32例（86.5%）。BMI范围位于17.1 kg/m^2^-30.8 kg/m^2^之间，中位BMI为26.3 kg/m^2^，27例（73.0%）患者的BMI超过25.0 kg/m^2^。所有病例的术式及诊断详见表 1，其中肺部疾病所占比例最多，为28例（75.7%）；恶性肿瘤34例（91.9%），比例远远高于良性疾病；开放手术共计22例（59.5%）。从发病时间来看，除1例术中大出血并行血管修补的胸腺癌患者怀疑术后发生PE外，其余36例患者在术后第1至13天内发生了PE，中位发生时间为术后第4天，其中发生在术后第3天的患者有11例，所占比例（29.7%）最高；另外，PE的发生时间在一天的24 h之内几乎均存在不同程度的分布，其中上午9点至晚上9点发生PE的比例可达77.8%（图 1）。在所有发生PE的患者中，13例（35.1%）患者在发病前曾有过排便病史（图 2）。发病时，37例患者均出现了不同程度的呼吸困难及气促症状，12例（32.4%）患者伴有咳嗽症状，5例（13.5%）患者伴发了瘫坐在地的一过性晕厥症状。37例患者的术后D-D波动在1.0 μg/mL-20.0 μg/mL（FEU）之间，平均值为（7.09±4.45）μg/mL（FEU）（图 3），其中32例（86.5%）患者的术后D-D高于3.00 μg/mL（FEU）。34例（91.9%）患者在发病第一时间所做的心电图上V1-V4导联出现了不同程度的T波改变以及ST段异常。治疗上，22例（59.5%）患者通过及时面罩吸氧及抗凝治疗后病情逐渐好转并最终出院。15例（40.5%）患者在发生PE后进行了气管插管机械辅助通气治疗，其中7例（46.7%）患者经积极治疗后逐渐脱机拔管并最终出院，剩下8例患者经插管上机及积极抢救治疗后无效最终死亡。

**1:** 37例的术后急性肺栓塞患者的术式及诊断情况 The diagnosis and operative procedure of 37 patients with postoperative acute pulmonary embolism

	Pulmonary disease		Mediastinal disease		Esophageal disease		Total
	Open	VATS	Open	VATS	Open	VATS	
Maliganant	13	13	4	1	3	0	34
Benign	1	1	0	0	1	0	3
Total	14	14	4	1	4	0	37
Open: open operation; VATS: video-assisted thoracic surgery.							

**1:**
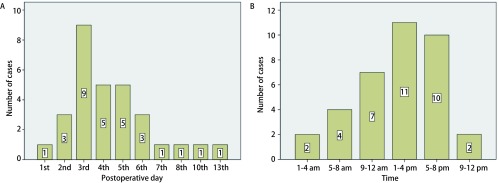
急性肺栓塞发病的时间分布情况。A：术后急性肺栓塞发病时间的分布情况；B：急性肺栓塞在24 h内不同时间点上分布情况。 distribution of acute PE in time. A: The distribution of onset time of acute PE in different postoperative days; B: The distribution of onset time of acute PE within 24 h.

**2:**
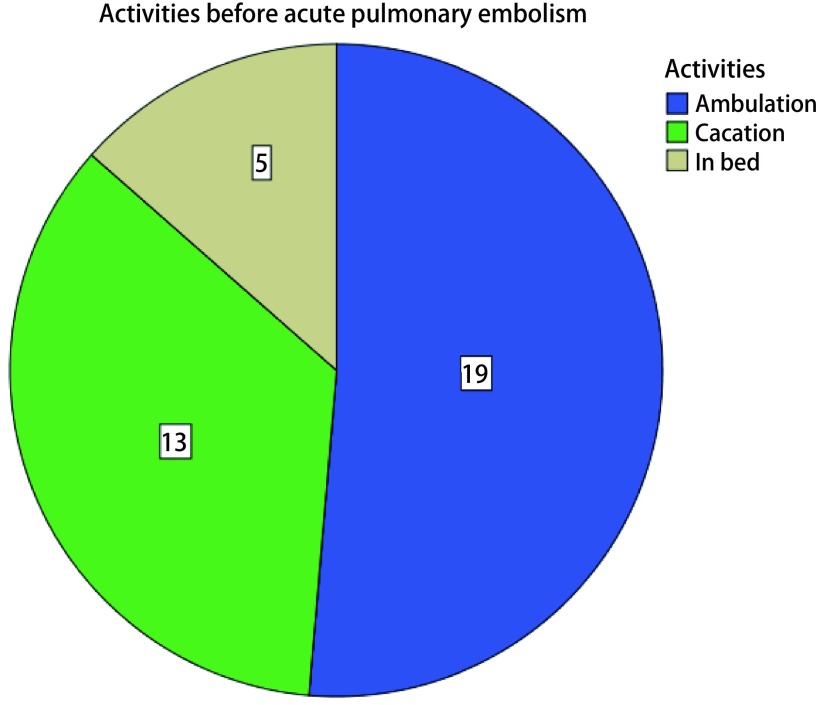
急性肺栓塞发病前患者活动情况 Activities before acute PE

**3:**
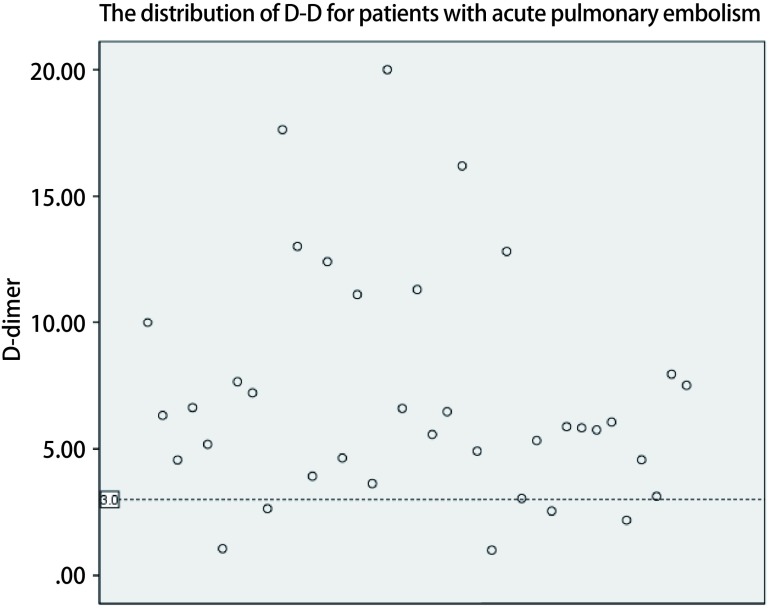
急性肺栓塞患者的D-D分布情况 The distribution of D-D for patients with acute pulmonary embolism

## 3 讨论

PE具有发生隐匿，误诊率高，病死率高的特点，近年来PE这种并不少见的术后并发症也得到了胸外科界的广泛重视^[6]^，欧洲胸科医师学会（European Society of Thoracic Surgeons, ESTS）于2016年成立国际工作组，并将2018年的工作目标确定为制定胸外科围术期VTE预防指南^[7]^。Singh等^[8]^曾指出胸外科术后PE的紧急处理至关重要，因为在发病后的几小时甚至几分钟内出现的延误诊治极有可能给患者带来严重的后果^[9]^。因此，如果能够对胸外科术后PE易患人群的特点有所了解，做到术后予以充分关注，出现问题及时解决具有至关重要的意义。

之前有研究对Caprini和Rogers等风险评估模型在筛选胸外科术后静脉血栓栓塞症（venous thromboembolism, VTE）的有效性上进行了验证^[10-12]^。Caprini风险评估模型作为美国胸科医师协会（American College of Chest Physicians, ACCP）抗栓与血栓预防实践指南第九版（ACCP9）中新增的针对非骨科手术患者的VTE风险评估量表^[13]^，包含了年龄、BMI、恶性肿瘤等约40个不同的血栓形成危险因素，通过这些危险因素对患者进行VTE风险评分。我们在本研究中也发现，胸外科术后发生PE的患者普遍具有老龄、BMI高以及伴有恶性肿瘤的特点。除上述危险因素外，长期卧床、包括肺炎（1个月内）在内的严重肺部疾病、中心静脉通路、静脉曲张、口服激素替代治疗、下肢水肿、VTE病史及血栓家族史等也均为VTE的危险因素^[13]^。此外，另有两篇病例报道描述了1例痛风性关节炎合并PE^[14]^以及一例肝硬化应用氰基丙烯酸异丁酯的硬化疗法^[15]^合并PE的患者，因此一些既往史中存在的良性疾病也应该引起足够重视。

本研究还发现，术后急性PE集中发生在术后第2-6天的范围内，并且相当一部分患者在发病前曾有过排便的病史。这提示我们对于高风险患者，在病情允许的情况下应尽可能让患者在术后前2天内开始下地活动，提早应用抗凝以及下肢间歇充气加压装置等能够降低静脉血栓栓塞发病风险的措施^[1]^，及时纠正排便障碍，同时排便后出现的突发呼吸困难对急性PE的诊断也具有提示作用。值得注意的是，虽然上午9点至晚上9点为急性PE的高发时间，但仍然有将近20%的病例发生在夜间9点至第二天上午9点之间，因此胸外科医师应尤其警惕可能发生于夜间的PE，以便发病时能够配备充足的人员及时进行抢救。另外，研究中大部分急性PE术后D-D超过了3.00，而对于肺癌患者，D-D在术后第1天-第7天内呈现升高趋势^[16]^，因此对于肺癌患者在术后常规监测D-D变化的过程中如果发现D-D超过了3.00 μg/mL (FEU)，需警惕患者可能已经形成VTE。此外，34例（91.9%）患者在发病第一时间所做的心电图上V1-V4导联出现了不同程度的T波改变以及ST段异常，这提示心电图对于PE的早期诊断也具有重要价值，尤其当合并右心功能不全时，心电图上常出现多导联T波倒置、S1Q3T3征（即Ⅰ导联S波加深，Ⅲ导联出现Q/q波及T波倒置）、右束支传导阻滞等改变^[17]^，3个及以上胸导联出现了T波倒置是PTE后肺动脉压力升高伴右心功能不全强有力的预测指标^[18]^。

对于已经疑诊PTE的患者，结合2018版PTE诊治与预防指南以及胸外科疾病诊疗过程的特点，其确诊及治疗流程详见图 4^[19]^。术后急性PE发病早期的诊断可以依据临床症状及体征、D-D及心电图表现进行初步判定，高度疑诊后应立即采取措施积极处理，包括卧床制动，面罩吸氧，必要时气管插管机械辅助通气。急性PTE临床高度疑诊后，建议病情允许时在等待诊断过程中开始应用普通肝素（unfractionated heparin, UFH）或低分子肝素（low-molecular-weight heparin, LMWH）等胃肠外抗凝治疗。病情平稳后应尽早完善金标准检查CTPA以明确诊断并指导后续治疗。在本研究中，29例患者通过CTPA确诊PE，其余8例患者在发病当时经抢救无效死亡，未来得及通过CTPA等检查确诊，因此临床诊断PE。确诊后应尽早启动抗凝治疗，如果选择华法林长期抗凝，需在应用胃肠外抗凝的同时重叠华法林，根据凝血功能逐渐调整华法林剂量，使INR维持在2.0-3.0之间停用胃肠外抗凝；如果选用利伐沙班或阿哌沙班，使用初期需给予负荷剂量，例如：利伐沙班15 mg日二次餐中口服3周，3周后改为20 mg日一次餐中口服。如果选择达比加群或依度沙班，应先给予胃肠外抗凝药物至少5 d。溶栓治疗只有在急性高危（即血流动力学不稳定）的PTE患者中推荐应用。

**4:**
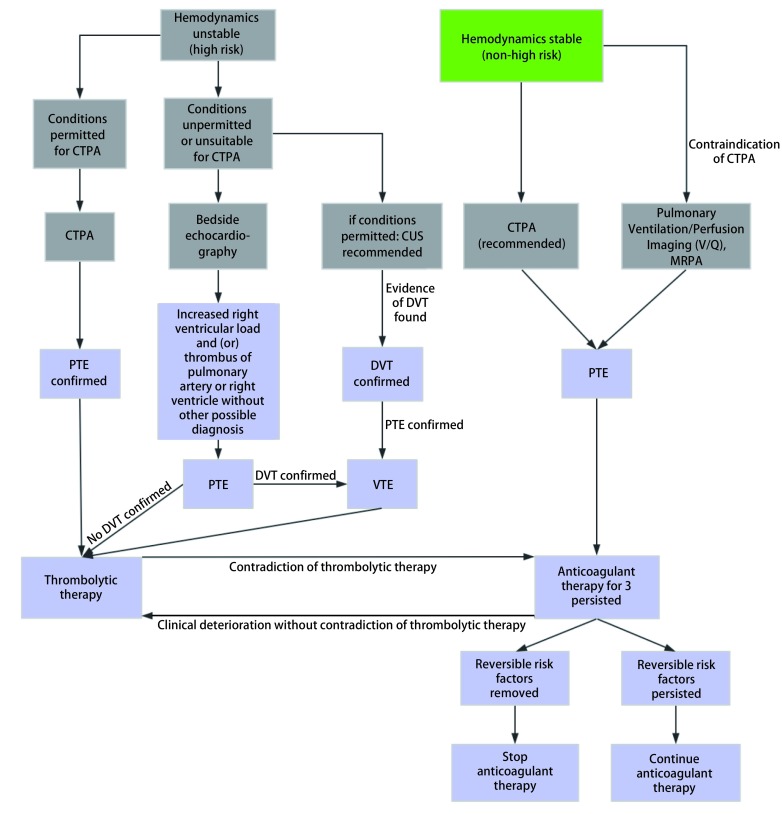
PTE患者的确诊及治疗流程 Flow chart of diagnosis and treatment for patients with PTE. CTPA: computed tomographic pulmonary angiography; CUS: compression venous ultrasonography; MRPA: magnetic resonance pulmonary angiography; PTE: pulmonary thromboembolism; DVT: deep vein thrombosis; VTE: venous thromboembolism.

综上所述，每名胸外科医师必须充分掌握术后PE的临床特点及诊治方法，及早发现诊断并采取多学科治疗能大大提高疾病的生存率。
